# Status of Oxidative Stress during Low-Risk Labour: Preliminary Data

**DOI:** 10.3390/ijerph20010157

**Published:** 2022-12-22

**Authors:** Barbara Zych, Anna Górka, Aleksander Myszka, Dominika Błoniarz, Aleksandra Siekierzyńska, Witold Błaż

**Affiliations:** 1 Institute of Health Sciences, Medical College of Rzeszow University, Warzywna 1a, 35-310 Rzeszow, Poland; 2 Department of Biotechnology, Institute of Biology and Biotechnology, College of Natural Sciences, University of Rzeszow, Pigonia 1, 35-310 Rzeszow, Poland; 3 Institute of Medical Sciences, Medical College of Rzeszow University, Warzywna 1a, 35-310 Rzeszow, Poland; 4 Department of Physiology and Plant Biotechnology, Institute of Agricultural Sciences, Land Management and Environmental Protection, College of Natural Sciences, University of Rzeszow, Cwiklinskiej 2, 35-601 Rzeszow, Poland

**Keywords:** oxidative stress, antioxidants, umbilical cord blood, full-term delivery

## Abstract

Pregnancy and childbirth are associated with the forming of reactive oxygen species that generate oxidative stress. Oxidative stress is a factor that may adversely affect the development of the fetus and the course of labour. Monitoring the parameters of oxidative stress can be used to assess the risk of health issues in the course of pregnancy and the condition of the newborn. Therefore, the analysis of oxidative stress in the physiological course of labour is the basis for understanding the role of oxidative stress in the pathogenesis of miscarriages and neonatal health circumstances. The study aimed to assess oxidative stress of mother-child pairs in the venous blood and umbilical cord blood at the time of physiological labour. One hundred and sixty-eight mother-child pairs were recruited to donate the mother’s venous blood in the first stage of labour and the venous umbilical cord blood after the newborn’s birth. Total antioxidant status (TAS), the activity of superoxide dismutase (SOD) with cofactors (Zn, Cu, Mn) and the activity of glutathione peroxidase (GPx) were analysed in venous blood plasma and umbilical cord blood. TAS value (*p* = 0.034), GPx activity (*p* < 0.001) and Zn concentration (*p* = 0.007) were significantly lower in maternal blood plasma as compared to neonatal umbilical cord blood. However, the activity of SOD (*p* = 0.013) and the concentration of Cu (*p* < 0.001) were significantly higher in the blood of mothers than of new-borns. The concentration of Mn in the plasma of the mother’s blood and the umbilical cord blood of the newborns was similar. Our research indicates higher levels of antioxidant enzyme (GPx) and total antioxidant potential (TAS) in umbilical cord blood compared to maternal blood, which may suggest depletion of redox reserves in women’s blood during labour.

## 1. Introduction

### 1.1. Oxidative Stress during Pregnancy and Childbirth

Physiological changes associated with pregnancy to meet the needs of the developing fetus and the health requirements of the mother may be accompanied by oxidative stress [1]. In the female, there is an increase in metabolism, greater oxygen consumption and the use of fatty acids as the primary energy source. Adapting the mother’s organism to the increased metabolic requirements of a 30-fold enlarging uterus, developing placenta and fetus requires large amounts of energy [2,3]. Rising blood volume increases the amount of oxygen, which may promote the formation of free oxygen radicals and, thus, lipid peroxidation [4]. The developing placenta with large amounts of mitochondria also stimulates the production of reactive oxygen species (ROS) [5]. ROS are oxygen-containing molecules with unpaired electrons and free radicals that act as oxydising agents by removing an electron or adding oxygen to other molecules [6]. Low concentrations of ROS perform physiological functions, and excessive levels of intra- and extracellular ROS can lead to irreversible cell damage, necrosis and apoptosis as a result of lipid peroxidation, protein changes, DNA damage and altered activity of membrane ion channels [4,6].

The oxidative stress arising during pregnancy is regulated by the body’s defence mechanisms, ensuring the balance between the production of free radicals and the antioxidant capacity, protecting the pregnancy’s course and the fetus’s development [7].

In the first trimester of pregnancy, hypoxia and oxidative stress are physiological conditions initiated by the maternal arterial circulation filling the interstitial spaces of the placenta. This condition predisposes to an increase in oxygen pressure, resulting in ROS formation and peroxides production [8,9]. ROS production occurs during the second trimester due to accelerated metabolism, with high oxygen and fatty acids consumption. During this period, the low flow, high blood resistance system shifts to a high flow, low resistance system. In the third trimester, there is an increase in insulin resistance, fat catabolism and the release of free fatty acids, increasing hydrogen peroxide production [10].

Moreover, the rising concentration of triglycerides, total cholesterol, LDL cholesterol and oxidative stress markers are associated with increased lipid peroxidation. At the end of pregnancy, nitric oxide synthase (NOS) activity in the uterus decreases [7,11].

The contractile action of the uterine muscle during labour reduces the uteroplacental blood flow. The changes start with the activation of the uterine muscle and increased production of oxytocin and prostaglandins [12,13]. During this time, vascular resistance decreases, and the diameter of placental vessels increases. This provides blood perfusion and protects the fetus against hypoxemia, and during delivery, against reduced uteroplacental blood flow [14,15].

### 1.2. Selected Antioxidative Mechanisms

**Figure 1:**
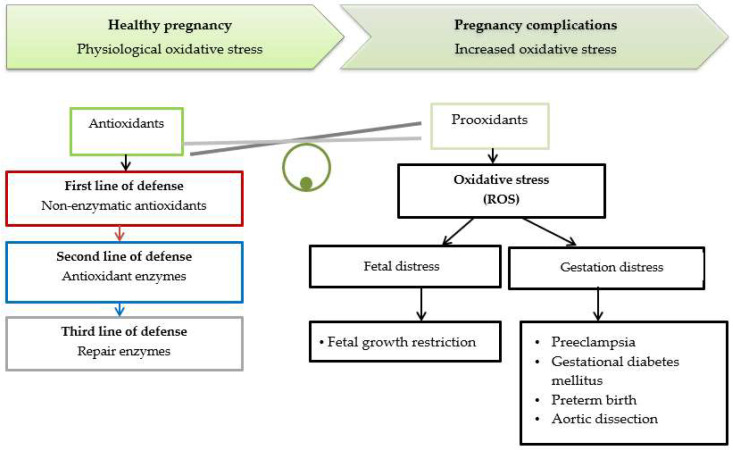
Clinical effects of oxidative stress imbalance during pregnancy.

Oxidative stress is an imbalance between reactive oxygen species (ROS) and the activity of antioxidants [10,16]. Excessive production of reactive oxygen and nitrogen species and insufficient antioxidants lead to the predominance of pro-oxidative processes (Figure 1). Extreme oxidative stress impairs maternal and placental functions and can cause much harm, leading to Intrauterine Growth Restriction (IUGR), preterm labour, gestational diabetes, preeclampsia and aortic dissection during pregnancy [17,18,19]. Although some oxidative stress is unavoidable in pregnancy, it is necessary to balance the production of oxidants and antioxidants [10,17], preventing oxidative damage and restoring redox balance [11]. Notably, the redox-sensitive Keap1-Nrf2 pathway associated with nuclear erythroid factor 2 (Nrf2) plays a crucial role in this process, which protects the fetus against oxidative stress [16,20,21]. Necessary in this respect is the adaptive cell defence system consisting of enzymatic antioxidants, including superoxide dismutase (SOD), catalase (CAT) and glutathione peroxidase (GPx) and non-enzymatic antioxidants e.g., α-tocopherol, coenzyme Q10, flavonoids, carotenoids and glutathione [17,22].

SOD catalyses the reduction of intracellular ROS through the dismutation of the superoxide anion (O_2_•−) to H_2_O_2_ which is dissociated into H_2_O and O_2_ by catalase. GPx reduces lipid hydroperoxides to alcohols and free hydrogen peroxide to H_2_O by using the reduced form of glutathione (GSH) as a hydrogen donor [14,16,23]. Antioxidant enzymes such as SOD and GPx increase gradually with the progress of pregnancy [24].

Manganese (Mn) is an activator of glycosyltransferase and other manganese-dependent enzymes such as arginase, phosphatase, choline esterase, and pyruvate carboxylase [25]. One of the most critical functions of manganese is its participation in antioxidant processes as SOD cofactor. The low manganese content in the diet has been found to influence the occurrence of anaemia [26], growth inhibition, neural tube defects, lower birth weight and psychomotor and mental disorders in children [26,27]. Additionally, manganese deficiency is correlated with increased lipid peroxidation and low birth weight of a child, which poses a risk of developing diseases in adulthood [28].

Zinc is part of the cytoplasmic superoxide dismutase and metallothioneins (proteins rich in cysteine residues that protect against free radicals). Being in the composition of the copper-zinc superoxide dismutase (Zn/CuSOD) participates in removing peroxynitrite formed in the reaction of superoxide anion with nitrogen oxide [5,29].

Copper is an integral part of the vital Cu/Zn-SOD antioxidant enzyme, and its deficiency lowers the activity of this enzyme. Copper deficiency affects the rate of catalase and Mn-SOD synthesis, which limits the body’s ability to eliminate ROS and leads to oxidative stress [27]. Copper has the ability to oxidise iron [30]. It participates in the synthesis of haemoglobin, which, although not an enzymatic protein, may exhibit catalase activity [31] and peroxidase [32].

Monitoring the total antioxidant status (TAS) can be a method of assessing the body’s ability to defend against oxygen-free radicals. Despite a significant increase in interest in the issues of oxidative stress and the antioxidant defence system in recent years, little attention has been paid in the literature to the changes occurring during childbirth. Therefore, our study aims to evaluate oxidative stress parameters in women giving birth and their offspring. For this purpose, we determined the total oxidation status along with enzymatic antioxidants, i.e., superoxide dismutase and its cofactors, and glutathione peroxidase in venous blood serum and umbilical cord blood. This article is an introduction to a broader analysis of the influence of oxidative stress on the course of pregnancy and childbirth and the health of the newborn, which will be presented in subsequent articles.

### 1.3. Aim

The study aimed to assess the oxidative stress of mother-child pairs in the venous blood and umbilical cord blood at the time of physiological labour.

## 2. Materials and Methods

### 2.1. Design

According to literature reports, oxidative stress may be a precursor of pathology in pregnant women, including eclampsia, miscarriage, preterm labour and intrauterine growth retardation. In offspring, it can lead to bronchopulmonary dysplasia, necrotising enterocolitis, retinopathy of preterm infants and periventricular leukomalacia [33]. Therefore, the assessment of oxidative stress parameters can be used to assess the risk of pregnancy pathology and neonatal diseases. Evaluating the parameters of oxidative stress in the proper course of labour is the basis for further research on the role of individual parameters of oxidative stress in the pathogenesis of pregnancy and neonatal diseases.

The issue of oxidative stress during low-risk labour and newborns is poorly understood, and there are few reports on this subject in the literature.

This study is part of basic research and contributes to extending knowledge about physiological values of oxidative stress parameters stress during low-risk labour.

Research on oxidative stress parameters in women during low-risk labour will be useful to establish reference ranges in women giving birth.

### 2.2. Data Collection

This research is a cross-sectional study.

The representative group of people was a random sample of pregnant women that agreed to participate in the project.

Data about participants were collected based on a survey among respondents, in the scope of the age of women, the number of pregnancies, weeks of gestation, delivery method, smoking and newborns’ condition.

The blood of women and umbilical cord blood were examined to find out the total antioxidant capacity, the activity of selected antioxidant enzymes, and the concentration of cofactors.

### 2.3. Biological Material and Its Preparation for Biochemical Analyses

The biological material consisted of pairs—samples of the mother’s venous blood and the newborn’s umbilical cord blood. The mother’s venous blood was collected from the elbow flexion vein in the first stage of labour (after qualifying the pregnant woman as giving birth), when contractions occurred regularly every 10 min, and internal examination confirmed anatomical changes in the vaginal part and the mouth of the cervix. Venous umbilical cord blood was collected from the umbilical vessel immediately after delivery. The material was deposited in 6 mL vacuum syringe tubes containing EDTA and lithium heparin. After collecting (up to 60 min), samples were centrifuged at a speed of 2500 rpm for 5 min (SIGMA 2-16PK) to separate plasma from blood cells. After centrifugation, the obtained plasma samples were stored at −80 °C. Before biochemical analyses, venous blood and umbilical blood samples were thawed and centrifuged for 1 min at 1000× *g*.

### 2.4. Oxidative Stress Analysis

According to manufacturer protocols, biochemical analyses were performed using commercially available kits from Randox (Randox Laboratories, Crumlin, UK). Absorbance measurements were conducted in 24-well plates using the Tecan Infinite M200 Pro plate reader and Magellan 7.1 software.

#### 2.4.1. Total Antioxidant Status (TAS)

Measurement of TAS in blood serum was performed by a colorimetric method using the kit NX 2332 (Randox Laboratories, Crumlin, UK). The principle of the method is based on the incubation of ABTS (2,2’-Azino-di-[3-ethylbenzthiazoline sulfonate]) with peroxidase (metmyoglobin) and hydrogen peroxide. The resulting ABTS radical-cation has a green-blue colour, the intensity of which is inversely proportional to antioxidant concentration [34]. Absorbance was measured at 600 nm. Randox Total Antioxidant Control (Cat. No. NX 2331) was used.

#### 2.4.2. Glutathione Peroxidase (GPx)

Determining the activity of GPx was conducted in accordance with the procedure of Paglia and Valentine [35]. GPx in the presence of cumene hydroxide catalyses the oxidation of glutathione (GSH). The glutathione (GSSG) formed in this reaction is reduced with the participation of glutathione reductase (GR) and NADPH, in this reaction, NADPH is oxidated to NADP+ [36]. The absorbance was measured at 340 nm after 1, 2 and 3 min.

#### 2.4.3. Superoxide Dismutase (SOD)

The activity of SOD was determined by measuring the degree of reaction inhibition method. Xanthine and xanthine oxidase were used to carry out the reaction in which superoxide radicals are obtained. Superoxide radicals by reacting with 2-(4-iodophenyl)-3-(4-nitrophenol)-5-phenyltetrazolium chloride, form the red formazan dye (I.N.T.). One SOD unit corresponds to the inhibition of 50% of the I.N.T reduction rate [37]. Results were obtained by measuring the absorbance at 505 nm after 30 s and 3 min.

### 2.5. Analysis of Selected Cofactors of the Oxidation-Reduction Reactions

Zinc, copper and manganese concentration in samples were conducted using ASA (Atomic Absorption Spectrometry). Samples were placed in a mineraliser vessel on the LA 230 S analytical balance (Sartorius) and the mass of the analyte was determined. Then 10 mL of Suprapur 65% nitric acid (HNO_3_) (Merck) was added to each vessel with plasma. The samples were incubated in the fume cupboard for 20 min for pre-digestion. Mineralisation was performed using an Ethos One pressure microwave mineralised (Milestone). Elements were determined using the Contr AA 700 spectrometer (Analityk Jena). Samples in the form of an aerosol in the plasma torch were excited and the intensity of the beam light, characteristic for a given element, were measured: for Zn—213.856 nm, for Cu—324.754 nm, for Mn—257.610 nm. The results were converted into µmol per liter of plasma and related to the CRM (Certified Reference Material).

### 2.6. Data Analysis

The data were analysed using the statistical program Statistica v.13 (StatSoft, Krakow, Poland). To indicate the existence of statistically significant differences between groups non-parametric (Mann–Whitney U) test (after checking test assumptions) was used. For evaluating correlations between analysed variables, Pearson’s correlation was used. The results for which the probability level was *p* < 0.05 were considered statistically significant.

The type of sampling was a random selection of pregnant women at the physiological delivery/appointed delivery date.

We assumed the variables under this study as the following:

- dependent: mother’s venous blood, umbilical cord blood,
- independent: total antioxidant potential (TAS), the activity of superoxide dismutase (SOD), the concentration of SOD cofactors (Mn, Cu and Zn), the activity of glutathione peroxidase (GPx).

### 2.7. Ethical Statement

All subjects gave their informed consent for inclusion before participating in the study. The study was conducted according to the guidelines of the Declaration of Helsinki and approved by the Institutional Review Board at the University of Rzeszow (protocol code No. 3/03/2015, date 25 March 2015).

## 3. Results

168 mother-child pairs were included in the study. The examined women were on average 30 years old (30.3 ± 5.44 SD). For most of them, it was the second pregnancy (65%). The majority of births ended by caesarean section (64%), at 39 weeks of gestation (39 ± 0.95 SD). Newborns were more often male (56%) with an average weight 3528 g (±435 SD) and body length of 56 cm (±2.82 SD).

Blood parameters of prepartum women were within the reference range: haemoglobin (HGB) concentration was 12.46 g/dl ± 7.01 SD, and the hematocrit (HCT) level was 36.03 ± 7.23). All participants were non-smokers.

We revealed a significantly higher (3.6%) TAS value in umbilical cord blood than in mothers’ veins blood (Table 1). The analysis of SOD activity showed significantly higher (4.4%) enzyme activity in the blood plasma of the women giving birth than in the umbilical cord blood (Table 1).

Mean concentrations of manganese in mothers’ blood plasma and umbilical cord blood of newborns were observed at a similar level.

We noticed more than five times higher (64%) copper concentration in maternal blood plasma compared to umbilical cord blood.

However, the zinc concentration in maternal blood plasma was significantly lower (11.2%) compared to umbilical cord blood.

The activity of the GPx enzyme turned out to be significantly higher (26%) in newborns’ cord blood plasma than in mothers’ blood (Table 1).

Among the analysed parameters, we revealed a weak correlation (r-Pearson = 0.3, *p* = 0.0002) between Cu and Zn in the mother’s blood. In turn, in umbilical cord blood, a negative correlation (r-Pearson = 0.35; *p* = 0.00002) between TAS and SOD was indicated.

## 4. Discussion

Pregnancy and childbirth are associated with increased production of reactive oxygen species, which may cause an imbalance between pro and antioxidants [38]. The parameter characterising the activity of the non-specific pool of antioxidants is the total antioxidant status, which determines the ability to protect against damage caused by reactive oxygen species and their derivatives [39].

Studies of pregnant women have shown that TAS level in the first trimester is significantly lower than in non-pregnant women. The dynamic of changes during pregnancy was also found in the second and third trimesters; the total antioxidant capacity of plasma (TAC) increases, while in the last week of pregnancy, it reaches values similar to those observed in non-pregnant women. After childbirth, this parameter increases to eight weeks after delivery [40].

In our study, the mean total antioxidant status (TAS) values were significantly lower in maternal blood than in umbilical cord blood, which may suggest depletion of the antioxidant reserve due to excessive production of reactive oxygen species during labour and reduced efficiency of the antioxidant system. Full-term delivery increases fetal antioxidant reserves [41], which may explain the observed differences between maternal and child TAS values.

An interesting observation was made by other researchers who assessed TOC (total oxidant capacity) that negatively correlates with TAS. In the umbilical cord blood, TOC was lower by about 38% compared with the maternal blood [42].

Decreased maternal total antioxidant status (M-TAS) may be associated with health disorders [42] resulting from changes in placental metabolism due to oxidative stress, protease activity, and factors involved in endoplasmic reticulum stress [43]. Moreover, higher antioxidants concentration at the end of pregnancy may be associated with a decrease in the levels of malondialdehyde (MDA) and nitrogen oxides (NOx) [7].

In our study, we observed a significantly higher superoxide dismutase (SOD) activity in the mother’s blood plasma compared to the umbilical cord blood of the newborn. In a study of other authors, the superoxide dismutase activity was found to be significantly higher (8.7%) in the cord compared to normotensive maternal blood; whereas, in the cord of preeclamptic maternal blood, the level decreased significantly [44]. In the study of third-trimester blood and the cord blood of mothers (smoking and non-smoking), they indicated higher activity of superoxide dismutase (CuZn-SOD) in cord blood [45].

In human organisms, a wide range of antioxidants prevent the formation of free radicals, inhibit their action or repair damaged molecules, protecting the body against their harmful effects [2,7]. Metal ions, i.e., manganese, copper and zinc, are bound with the regulatory function, the synthesis of hormones and act as cofactors of antioxidant enzymes [46]. During childbirth, they balance oxidative stress and regulate the inflammatory response in the placental membrane by releasing the nuclear factor kappa B (NF-κB) [47]. Metal ions’ disproportion between the mother and the fetus could lead to different profiles of the oxidative response and changes in oxidative and antioxidant markers [7,48].

In our study, mean manganese concentrations in venous blood plasma and umbilical cord blood were similar. Takser et al. [49] showed a gradual increase in Mn in the peripheral blood of women along with the development of pregnancy and that the concentration of this element in the umbilical cord blood is higher than in the mother’s blood [26,49]. Similar observations were made by Yazbeck et al. [50], who found a correlation between the manganese concentration in the blood of mothers and newborns. The cited studies [49,50] suggest the existence of a mechanism of active manganese transport from mother to newborn [51], resulting in a higher concentration of Mn in the umbilical cord blood than in the maternal blood [52]. However, the results of our research did not confirm this observation.

Zinc also plays an essential role in the defence system against ROS. Our study noticed a significantly higher zinc concentration (Zn) in the umbilical cord than in the mother’s blood. This phenomenon may be the result of active placental zinc (Zn) transfer and the accompanying endocytic mechanisms [53], maintaining a higher concentration of the element in the placenta than in the mother’s body [54]. The human organism does not have zinc reserves; the only exception is newborns born at term. Eutrophic newborns can derive a reserve of zinc accumulated at the end of pregnancy from their liver, which is mature enough to store trace elements [47]. Therefore, zinc deficiency in the fetus is rarely observed, only in severe deficiency of this element in the mother [54]. Although the studies of other authors indicate that the concentrations of trace elements in the blood are very diverse, the concentrations of Zn and Cu are generally high [54,55,56]. Comparing our results with the results of other researchers, we also noticed that the population of Polish women was characterised by a similar concentration of Zn and Cu as compared to Chinese women [57], but a higher concentration of both micronutrients than in Italian women [55] and a lower concentration of Zn than Australian women [56]. The results of our research have shown that the level of Zn in the plasma of venous blood and umbilical cord blood of a newborn is consistent with the results of other Polish researchers [58].

The copper concentration in mothers’ venous blood was twice as high as in other studies [58]. The discrepancy in the results may be related to the living environment, the type of diet, dietary supplements, and the pregnancy sequence [57]. In addition, in the studies by Chiudzu et al. [47], women in physiological pregnancy showed high copper (Cu) concentrations in the mother’s serum and were not associated with an increased risk of preterm labour. However, according to other researchers, the increased zinc concentration in the mother’s blood increases the risk of preterm delivery [47].

In the material we studied, we noticed that the concentration of copper was 20% lower in the plasma of the umbilical cord blood compared to the mother’s blood. A possible cause of this situation may be the blocking copper ions at the placenta [58,59]. The team of Kot [58] made similar observations to ours, showing 50–60% lower concentration of the tested element in the umbilical cord blood compared to the maternal blood.

Additional body protection against peroxidative damage constitutes the haemoglobin; concentrations in women studied in our research were in the reference range.

We also analysed glutathione peroxidase activity (GPx), which mainly protects cell membranes against peroxidative damage [60,61,62]. The activity of the GPx enzyme in the umbilical cord blood was significantly higher than in the mother’s blood by about 26%.

Other researchers also revealed that GPx activity was significantly higher (about 16.4%), in the cord than in normotensive maternal blood. In preeclamptic, changes in GPx activity were not significant [44].

The results of other authors indicated that in the group of smoking mothers, the extracellular form of glutathione peroxidase (GPx-3) level was significantly higher than observed in cord blood. Contrasting, in the non-smoking group, the GPx-3 concentration did not differ between mothers and newborns [42]. However, in our study, we tested the activity of total GPx, while Chełchowska [42] tested the level of an extracellular form of glutathione peroxidase (GPx-3). Studies by Khan et al. [63] showed a reduced expression of GPx protein in the blood of women with full-term delivery compared to non-pregnant women. Abiak’s team made different observations [64]. They showed a lower level of GPx activity in newborns’ umbilical cord blood serum compared to their mothers. The team suggests that the decreased activity of GPx is most likely because the enzyme is the primary cytoprotective enzyme that requires superoxide dismutase substrates [64].

Scientific studies suggest the role of diurnal regulation of protein expression in the cellular response to oxidative stress. One of the critical groups of antioxidant enzymes sensitive to daily expression turns out to be SOD and GPx [65]. Stritesky Larssen et al. confirmed this relationship by observing the variability of ROS production in leukocytes along with the circadian rhythm, demonstrating a higher antioxidant status in newborns as opposed to adults [66].

Each marker of oxidative stress has both advantages and disadvantages. The control of oxidative stress parameters in the blood has limitations due to invasive methods. Therefore, other methods of assessing the severity of oxidative stress may also be helpful.

Lastly, research revealed that markers of oxidative stress in urine: 8-iso-prostaglandin-F_2α_, 2,3-dinor-5,6-dihydro-8-iso-prostaglandin-F_2α_ (F_2_-IsoP-M—the major metabolite of 8-iso-prostaglandin-F_2α_) and prostaglandin-F_2α_ are associated with increased risk of spontaneous preterm birth [67].

Other researchers revealed that exposure to air pollution (PM2.5) increases oxidative stress in pregnant women and is associated with early pregnancy loss [68]. Monitoring air quality and avoiding exposure to high air pollutants can prevent increased oxidative stress and pregnancy complications.

Analyses of oxidative stress in pregnant women should include a wide range of oxidative stress biomarkers in particular trimesters, which may allow clinicians to identify the risk factors and monitor them during pregnancy to direct the activities to include potential therapies, e.g., progesterone supplementation [67].

## 5. Conclusions

Evaluation of oxidative stress enables the determination of the degree of cell damage and the risk of developing associated diseases. Directly analysing the amount of reactive oxygen and nitrogen species is a difficult task; therefore, in assessing the severity of oxidative stress, compounds formed as a result of ROS reactions with cell components, antioxidant capacity and the activity of antioxidant enzymes are more often taken into account. The share of trace elements, which are components of enzymatic defence, is also essential in oxidative stress response. In normal pregnancy and physiological labour, when oxidative stress is increased, the antioxidant defence mechanisms can counteract this phenomenon through enzymatic induction and activity and with the participation of non-enzymatic protectors and free radical scavengers. However, it should be noted that pregnancy is a state in which the body’s adaptation and balance can be quickly disturbed. Therefore, it is crucial to look for markers that we can monitor with readily available and quick diagnostic tools to determine the potential risk of disorders and counteract them.

This study provides information on the physiological range of oxidative stress parameters in the mother’s veins blood and umbilical cord blood during low-risk labour.

The results of our research indicate differentiation in total antioxidant capacity, antioxidant enzymes activities and trace elements between the blood of the mother and the child in the conditions of physiological delivery, which prompts the need for further analysis of these parameters in various disorders in the course of pregnancy and childbirth and in the health condition of newborns. These analyses may allow for the identification of early markers of the risk of preterm labour and diseases of the newborn.

Research on oxidative stress parameters in women during low-risk labour will facilitate establishing reference ranges in women giving birth. Further studies may also contribute to the development of laboratory/diagnostic tests to assess the risk of childbirth complications and health disorders of the newborn.

Currently, there is no ideal marker for assessing oxidative stress in pregnant women; therefore, investigating different parameters and searching for new especially based on non-invasive methods is crucial.

## Figures and Tables

**Table 1:** Parameters of oxidative stress in the mother’s venous blood and the newborn’s umbilical cord blood.

Parameters	Mother’s Venous Blood (n = 168)			Umbilical Cord Blood (n = 168)			*p* Value
	$\bar{x}$ ± SD	Median	Min-Max	$\bar{x}$ ± SD	Median	Min-Max	
TAS [mmol/L]	1.65 ± 0.54	1.66	0.53–3.30	1.78 ± 0.37	1.76	0.65–4.00	0.034
SOD [U/mL]	205.02 ± 38.46	210.18	144.37–266.77	192.40 ± 29.28	194.07	132.24–281.41	0.013
Mn [µmol/L]	0.11 ± 0.24	0.06	0.01–2.87	0.11 ± 0.13	0.08	0.00–1.11	0.079
Cu [µmol/L]	32.86 ± 7.85	32.30	5.00–77.40	7.17 ± 4.35	6.60	0.50–33.40	0.000
Zn [µmol/L]	12.13 ± 8.50	10.75	1.00–58.50	15.17 ± 13.60	12.40	2.00–143.9	0.007
GPx [U/g Hg]	95.14 ± 42.97	88.49	2.81–211.40	151.13 ± 96.77	141.87	93.41–1057.14	0.000

TAS—total antioxidant status, SOD—superoxide dismutase, Mn—manganese, Cu—copper, Zn—zinc, GPx—glutathione peroxidase, *p*-value—the probability of Mann-Whitney test.

## Data Availability

The data analysed in the study are available upon request to the corresponding author.
